# Supplementary material for: NOVA-dependent regulation of cryptic NMD exons controls synaptic protein levels after seizure
Source: eLife. 2013 Jan 22;2:e00178. doi: 10.7554/eLife.00178 (PMC3552424; doi:10.7554/eLife.00178)
Supplement: Figure 3—source data 1. — DOI: http://dx.doi.org/10.7554/eLife.00178.012 [file elife00178s001.doc]

Figure 3 Source Data 1

# Sequence of RT-PCR products (From Figures 3 and 5)

## Dzip1 (upper band in DKO)

GCTCGACTATGAGCAGAGCAAGCAGCTGCTCACCAAGCAGGCTGGCGAGATCAAGCTGCTGAAGGAGGAGTGTAAACGCAGGAAGAAGATGCTGTCCACGCAGCAGCTGATGATAGAGGCCAAGGCCAGCTATTACCAGTGCCATTTCTGTGACAAGGCCTTTATGAACCAAGCGTTCCTACAAAGCCACATCCAGCGCCGCCATACTGAAGACTCTCACCTCGGTCATCTT**TGA**GATTCTGACTTTCTGCTTTGGTTCCCATACCTTTCATGTCTACACTCACAGCACATGTATACCATTCAGTCATGTGTACATCCTCGTAGTATAATACAAAGGCCCAGACCGACAGGCTCCAGAAGGAGATTGACATGTTGAAGGAACAGCTGCAGCTCACTAGGTCTCAGCTGGAGTCTGCACAGCACTCTCATGCAGTTCGCTTCTCTAAGGACTATGAAATGCAGAAATCAAAAGAGGAAGACTTTCTGAAGCTGTTTGACAGATGGAAAGAGGAGGAGAAGGAGAAGTTACTCGAGGAGATGGAGAAGGTCAAGGGGATGT

## Slc4a3 (upper band in DKO)

AAGACCTTGGCTGTGAGCAGGTTTGGGGACCTCATCAGCAAGACCCCGGCCTGGGACCCCGAGAAGCCTAGCCGCAGCTACAGCGAGCGGGACTTTGAGTGTGGG**TAG**CCTGAGGACCCCTAGCACCCCAGCCTTCACCACCATCACCTTCATCGCCACCATTACTGCGCTCACCTCCGGCTTGATCACTCAGTGTCATCCTGTGCTGGACGCTGTGCTGGGCCACCATGCCATGTTCACCGGCACACATCCCACCACACCCATCACCCGCTCTCAGCCCGCCTGCCTCCACCCCACAAGCTTCGGCGACCGCCCCCCACCTCTGCTCGGCACACCAGGAGGAAGAGGAAGAAAGAGAAAACCTCTGCTCCCC

## Rasgrf1 (one of upper bands in DKO)

AGCTCCAAAAGCTTGTGTCATCAGATGGCAGATTTAAGAACCTCAGAGAGTCTTTGAGAAAACAGATGTTGAACCCATCTGTGCTATCCGGCCGTCTCTCCTCCGCCCGGGCTCCCTGGAGCCCTTGTGGACTGTGCTTATACGAAGCTGCCCCTGTGCATTTACCAACTGTCCGTCTGCCTCTGGGCGTTGCTCCATCTGCCTCCTTCACACGGCTTCCTCATCCACTGCAACCACTACTGCTACCTAACTGTCTCCGTGGCTCCCATTCTGTC**TGA**CCCTCTCCTGACTGTCTTCCCTTTCAGGTCACCTGTATGCCTGTTTGTGATCCACCCTGTGTCCCTTACCTGGGGATGTATCTCACCGACTTGGTGTTCATCGAGGAGGGGACACCCAATTACACAGAGGACGGCCTG

## Cdk5rap2 (upper band in WT)

AAGGTTTCTCCCACCAGAGCCCGGGACATGAAGGACTTTGAAAACGGTTGCACACTTCCTTTGGTATCATTTCCTTTCAATCTGAAGAACTTCCTCTGTTGTTTCCTT**TAG**AGCAGCAAATCACTGAGCTGAAGAAGGAGAACTTCAATCTAAAGCTCCGGATCTACTTCCTGGAGGAGAGAATCCAGCAGGAGTTTGCTGGCCCCACCGAGCACATCTACAAGACGAACATTGAACTCAAGGTGGAAGTGGAAAGTCTGAAGCGCGAGCTCCAGGAGAAAGATCAGTTGCTCGTCAAAGCCTCCAAAGCGGTGGAGAGCCTAGCAGAACGGGGTGGTTCTGAAGTCCAGCGGGTGAAAGAAGATGCAAGGAAGAAGGTGCAG

## Slc4a10 (upper band in DKO)

GAAGCAGCATCATCACCAGAATCAGAAAAAACTGGCTAACAGGATTCCTATTGTCCGATCTTTTGCTGATATTGGCAAGAAACAATCAGAACCAAATTCCATGGATAAAAATGGCTCATCTCATCTCTGTACCTCCCTCATCTCCTACCCTTTGGGTACTCTAAATGCGTTGAAGGTTCTGAGGTCAGGTTGTTTCTCCTCAGTCTGCTCCAGCCTGTGC**TGA**GAATAAAAATGATGTCAGCAGGGAAAACAGCACTGT

## Plekha5 (middle band in WT)

TGGCAGGATCAAAGCCTTTCTCATCAGTTAAGTACAAGAGCGAGCAGGGGCCCCGTGCATCTGCC**TGA**AGACAAGAAGATTCATCAAGTTCAAGGATATCCAAGAAATGGATCTCACTGTGGTCCAGATTATAGACTCTACAAGAGTGAGCCAGAGTTAACCACAGTGGCAGAAGTCGATGAATCTAACGGAGAAGAAAAGTCAGAGCCAGTTTCTGAGACAGAAGCTCCAGTCG

## Ahi1 (upper band in DKO)

CCCTCCTTTAACTCCCAAGGAGAAAACTAAACCAGAAAAGCCTCTGGCTTCTCAAAAGGGTGGCCATGAAGAGGAGACGAAGTCACAAACCAAC**TGA**GAGGTGTGTGTGGAAAGGAGGGAAGGAAGGGCCGGCTGCCATGCTCCTTGGCCTCGCTGATGGTGGGGGGGGCACTTGTGGGCTGATTGCGGTCCAGCCGTGGGGGCTTTTGTTTAAGATAAAATAAAACAGCTGACAGAGACAACTCCTTCTTCCCCCTCCTACTGCCTCCATGAGCACTTCCCATATGGCTGCTGCTGCTGAGACCAAGGAATGACAGTCGCTCAGTAAGGGCAGACCCCTGGATCCCAGACTGGGCCCGCAGCCTGTGGGGCATTCTGAGAAGGGCAAAGATCAAAACGTGGAGGACCGAGGACACAAAGTAGATATGGAGACAAAGAAAAGCGAGCCGGTGGTCCGCAAAGTCACCCTGATAGAGTAAAAAGCTGGAGACGAAGCAGAAGCCGCCTGATGAGTGCACATGATGTGACGTCCACGCCAGACGGAGTTCTCGTTAGATTTTGGGTGAAAAGAAGCAGTATTTATTGACCTTAAAAATCTGAAGCAAACTTAGCTCTAAAAAGAAAAAAATCACTGTGGCCTTTGG

## Actl6b (lower band in DKO)

GCAGCGTAGAGAGGGCACTATGAGCGGGGGCGTCTACGGCGGAGGTGAGCCGGCCTGGGGCACTGGGCTCTCGGCCTGGTCTACAGCCCTGCATCCCGAACCCGCGCGTCCCAAGCCC**TGA**GCATCCCTGGGCCTGTCACCTCCGTACTGCATCAGGTCCAGTCTGAGTGGAACACTCGGGCCAAGCGGGAGAAGCTGACGGAGCTGATGTTCGAGCAGTACAACATTCCTGCCTTCTTCTTATGCA

## STX2 (upper band in WT) (supplementary Figure 5)

TGCACGAGATGTTCATGGATATGGCCATGTTTGTCGAGACTCAGACTCTGTCTCCTCCTGGACGGGACCCAGGG**TGA**AATGGTCAACAACATCGAGAGAAATGTGGTGAACTCTGTAGATTACGTGGAACATGCCAAGGAAGAGA

## Dlg3 (upper band in DKO) (Figure 3)

CCAGGACAGGGATGATTGAGTCTAATCGGTCGATCAAAACGAAACGTAAAAAGAGTTTTCGCCTCTCTCGAAAGTTTCCATTTTACAAGAGCAAAGAAAACATGGCCCAGGAGAACAGCATACAGGAACGACTTCCCTGGGT**TAA**GTGACGATTATTATGGAGCAAAGAACCTGAAGGGAGTGACATCCAACACCAGTGACAGCGAAAGCAGTTCCAAAGGACAAGAGGATGCTATTTTGTCATATGAGCCAGTGACACGACAA
