# Supplementary material for: NOVA-dependent regulation of cryptic NMD exons controls synaptic protein levels after seizure
Source: eLife. 2013 Jan 22;2:e00178. doi: 10.7554/eLife.00178 (PMC3552424; doi:10.7554/eLife.00178)
Supplement: Supplementary file 2. — PCR primers used in this work. All PCR primers are shown, oriented 5′ to 3′. DOI: http://dx.doi.org/10.7554/eLife.00178.029 [file elife00178s003.docx]

# Supplementary File 2: PCR primers used in this work

All PCR primers are shown, oriented 5’ 🡪 3’.

qRT-PCR primers

| Qsap102_5 | GGCAGACATACGAACAAGCA |
| --- | --- |
| Qsap102_6 | CCCAGACTGGTCCTCAATG |
| Qdzip1_1 | CCTCGGGAATTCAAGGAAG |
| Qdzip1_2 | AGGACATCGCTCCAGTCAGT |
| Qdtd1_23 | TTCGCAAGAGCTACAGACCA |
| Qdtd1_24 | ATAGTCACAGGCCCATCGTT |
| Qrasgrf1_51 | TCGCCAGTTTCAGCAGACTA |
| Qrasgrf1_52 | CTCATGTGGGGAGTTTTGGT |
| Qslc1a6_53 | CCACAGAGGACATCACACTGA |
| Qslc1a6_54 | CCCCCAGTACATTGGTCATT |
| QRasgrp2_55 | GACGGAGTGTTTGACATCCA |
| QRasgrp2_56 | CAGAATCTCTGGCTCCCTTC |
| Qslc4a10_5 | AAGGAGTCACGCTTTCCTTC |
| Qslc4a10_6 | CCTTTCCCTGAGTCAGCTTT |
| QAhi1_7 | GAGGACCGAGGACACAAAGT |
| QAhi1_8 | TCTGCTTCGTCTCCAGCTT |
| Qly6_25 | GCCATGGCCACCTACTGTAT |
| Qly6_26 | CGTACACGGTTTCAAAGCAG |
| Qcdkl1_31 | ACAAACCAGCGAGGAAGACT |
| Qcdkl1_32 | TGCTGGTTAGCTGAGGTAGG |
| Qrwdd2a_29 | AGGTATCTGGAAGGCACCAG |
| Qrwdd2a_30 | GCCACGTATGGGTAGTTGTG |
| Qvamp1_11 | AGCAGTGCTGCCAAGCTAA |
| Qvamp1_12 | ACTACCACGATGATGGCACA |
| Qsyt2_19 | ACGAGTCCTTCAGCTTCGAG |
| Qsyt2_20 | CGTAGTCTAGCACGGTGACG |
| QCacna2d3_3 | AGAAGATCAGACGACGTCCAG |
| QCacna2d3_4 | GCCTGAAGACTCGATGCAC |
| Qcpne3_15 | GGAGAGGTGGCCATAAGAGA |
| Qcpne3_16 | GCCAAGACACACTGAGCAAG |
| Qstx2_27 | AAGCCGTCCATCTTCATCTC |
| Qstx2_28 | ATGAACATCTCGTGCAGCTC |
| Qscn9a_41 | CTTCACTGTGGGATGGAACA |
| Qscn9a_42 | CGGAACAGGGTAGGAGACAC |
| Qplekha5_45 | CTTGAAGAAGAGCCCAGAGG |
| Qplekha5_46 | GGAGTGTCCGGTAACCTTCA |
| Qstxbp2_47 | TCGTGTACATTGTGGGTGGT |
| Qstxbp2_48 | AAGCGGGTTGGAGTAAGGAT |
| Qsyngr3_33 | GCTTCTTCTCCATCCTCAGC |
| Qsyngr3_34 | CCTAGCTGATCTGTGGCAAA |
| Qglrb_5 | CAGATGGGAAAGGTGGAAAC |
| Qglrb_6 | TTGCATCTGGTCTCACCAAC |
| Qgabbr1_9 | AGTCCCGACTGTTGGAGAAG |
| Qgabbr1_10 | CAGTTCAGAGACACGCTCCT |
| Qslc4a3_41 | GCACTTCTCTGGGTGGTCA |
| Qslc4a3_42 | CCGCATCTTCAGAGTCCAG |
| Qupf1_3 | GCCTGCAGTTACTGTGGAATC |
| Qupf1_4 | TAGCACTCCAGCACGGTCT |
| qd1EGFP_F | GATCACTCTCGGCATGGAC |
| qd1EGFP_R | TCTCCTGGGCACAAGACAT |
| Qneomycin_13 | CTCGACGTTGTCACTGAAGC |
| Qneomycin_14 | ATACTTTCTCGGCAGGAGCA |

RT-PCR primers

| Lrrcc1_1 | GTGTGTAGCGAAATCGAACG |
| --- | --- |
| Lrrcc1_2 | TCAGATTAACCAGTGCTTCAAGT |
| Ahi1_1 | CCCTCCTTTAACTCCCAAGG |
| Ahi1_2 | CCAAAGGCCACAGTGATTTT |
| dzip1F | TTCCTACAAAGCCACATCCA |
| dzip1R | ATTCCCTCATAAACATCCCC |
| SAP102_36 | CCAGGACAGGGATGATTGAG |
| SAP102_37 | TTGTCGTGTCACTGGCTCAT |
| Stx2_16 | TGCACGAGATGTTCATGGAT |
| Stx2_12 | TCTCTTCCTTGGCATGTTCC |
| Slc4a10_11 | GAAGCAGCATCATCACCAGA |
| Slc4a10_12 | ACAGTGCTGTTTTCCCTGCT |
| Slc4a3_5 | CAGGTTTGGGGACCTCATC |
| Slc4a3_6 | ACAGGCTCTGCCTCAGACTC |
| SCN9a1_1 | TCACAGTGACAGTGCCCATT |
| SCN9a1_2 | TCAGCTTCTGCCTCCTCTTC |
| STXBP2_1 | CTGAAGGCGGTGGTAGGG |
| STXBP2_2 | CCGTGGGACTCAGCAAATAA |
| Rasgrf1_1 | AGCTCCAAAAGCTTGTGTCAT |
| Rasgrf1_2 | CAGGCCGTCCTCTGTGTAAT |
| cdk5rap2_1 | AAGGTTTCTCCCACCAGAGC |
| cdk5rap2_2 | CTGCACCTTCTTCCTTGCAT |
| PLEKHA5_5 | GGCAGGATCAAAGCCTTTCT |
| PLEKHA5_6 | CGACTGGAGCTTCTGTCTCA |
| actl6b_1 | GCAGCGTAGAGAGGGCACTA |
| actl6b_2 | TGCATAAGAAGAAGGCAGGAA |

PCR primers for constructs

| DLG3_13 | ACATCTCGAGCTTAAAAAATGTTGTTTTGTTTTGGTTTCTCTTTCG |
| --- | --- |
| DLG3_4 | ACAATGGAAAGACAGACTCTGGACAACTGCACAGCTTGTGAAGCCGCGG |
| SAP102_13/-r | GGGATGATGCGGTCTGGCTGGTGGCGTGACCGTGCCGGTTCCTGCATG |
| DLG3_18_YCAY | ACACTCGAGCATGATGCCATCTCACTCATCA |
| DLG3_18_YAAY | ACACTCGAGCATGATGCGGTCTGGCTGGTGG |
| DLG3_19 | ACACCGCGGCTTCTGCTGGGTGTTTAT |
| SYT2YCAY_1 | ACATCTCGAGCTTAGTATGTGCCCATCCATCCCCATCTGCC |
| SYT2YCAY_2 | ACACCGCGGCTTGCTTTCCCTTGGAGTCTCTGAAGACA |
| SYT2YAAY1_1/-r | TCTCTTAATTTTCTGGAATGAGTAATGGACAGTGAAGCAATGTGAG |
| SYT2YAAY1_2/-r | GTATGTGCCAATCAATCCCAATCTGCCTGTTTTGAACATCTCTCT |
| GlrbYCAY_1 | ACATCTCGAGCTTGATAAACCATTTCCCTTTGTATGA |
| GlrbYAAY_4 | ACATCTCGAGCTTGATAAACAATTTCCCTTTGTATGA |
| GlrbYCAY_2 | ACACCGCGGCAATATATTGCTAGGAAAATGCAAAG |
| GlrbYAAY_3/-r | CGAGCTTGATAAACAATTTCCCTTTGTATGAAATACAGCA |
| Glrb7/-r | GAAATACAGCAACATTTCAGTGTGACCAATGCAATTAATAATTGCTGA |
| Syngr3YCAY_1 | ACATCTCGAGCTTAGTCTATCTTCTATCCCCTTTTAAGGC |
| Syngr3YCAY_2 | ACACCGCGGCATTTTGTCTGGAAGAATGAGACAT |
| Syngr3YAAY_3/-r | AGCAGACTACTAATACATTGCTTGAAGCTGGCTTTTAACATCAAG |
| Syngr3YAAY_4/-r | TCAAAACCAAACGCGTTGCAATGTCTAATTCTTCCAGACAAAATG |
| Syngr3_3 | TCCTTCCTGTGGTTCGTAGG |
| Syngr3_4 | CGGTAGGACCCAGAAAAGGT |
